# How should we define a nociceptor in the gut-brain axis?

**DOI:** 10.3389/fnins.2022.1096405

**Published:** 2022-12-19

**Authors:** Nick J. Spencer, Tim Hibberd, Zili Xie, Hongzhen Hu

**Affiliations:** ^1^Visceral Neurophysiology Laboratory, College of Medicine and Public Health, Flinders Health and Medical Research Institute, Flinders University, Bedford Park, SA, Australia; ^2^Department of Anesthesiology, The Center for the Study of Itch and Sensory Disorders, Washington University School of Medicine, St. Louis, MO, United States

**Keywords:** pain, nociception, spinal afferent, visceral pain, colon

## Abstract

In the past few years, there has been extraordinary interest in how the gut communicates with the brain. This is because substantial and gathering data has emerged to suggest that sensory nerve pathways between the gut and brain may contribute much more widely in heath and disease, than was originally presumed. In the skin, the different types of sensory nerve endings have been thoroughly characterized, including the morphology of different nerve endings and the sensory modalities they encode. This knowledge is lacking for most types of visceral afferents, particularly spinal afferents that innervate abdominal organs, like the gut. In fact, only recently have the nerve endings of spinal afferents in any visceral organ been identified. What is clear is that spinal afferents play the major role in pain perception from the gut to the brain. Perhaps surprisingly, the majority of spinal afferent nerve endings in the gut express the ion channel TRPV1, which is often considered to be a marker of “nociceptive” neurons. And, a majority of gut-projecting spinal afferent neurons expressing TRPV1 are activated at low thresholds, in the “normal” physiological range, well below the normal threshold for detection of painful sensations. This introduces a major conundrum regarding visceral nociception. How should we define a “nociceptor” in the gut? We discuss the notion that nociception from the gut wall maybe a process encrypted into multiple different morphological types of spinal afferent nerve ending, rather than a single class of sensory ending, like free-endings, suggested to underlie nociception in skin.

## Introduction

Nociceptors are primary afferent neurons that are capable of detecting and responding to potentially damaging stimuli (Kandel et al., 2000). In the skin, different types of sensory nerve endings have been well characterized (Handler and Ginty, 2021) and cutaneous nociceptors are defined as a unique class of afferent fiber that is morphologically considered a “free-ending” (Kandel et al., 2000). Unlike the skin, however, where the functional role of the different morphological types of sensory nerve endings are well characterized, our understanding of the functional role of the different morphological types of extrinsic sensory nerve endings in visceral organs, like the gut is limited. Even less clear is which sensory nerve endings in the gut encode nociceptive stimuli.

A variety of stimuli can activate sensory nerve endings from within the gut wall which initiate the sensation of pain. The visceromotor response (VMR) to colorectal distension (Castro et al., 2013; Fuentes et al., 2016; Kyloh et al., 2022) or bladder distension (Zagorodnyuk et al., 2011) is commonly used as a model for visceral pain measurements. The VMR is a complex neural reflex response from the visceral organ to the central nervous system (CNS). The motor response is contraction of the abdominal muscles (Laird et al., 2001). Mutant mice lacking the TRPV1 channel have reduced VMR distension-evoked pain sensations following colorectal distension (Jones et al., 2005). Hence, there is sound evidence to suspect that Trvp1 expressing spinal afferents encode nociceptive stimuli from the gut to the CNS.

## Nociception in the gut

There is no doubt that in vertebrate animals, noxious or potentially damaging stimuli applied to the gut can trigger the sensation of pain (Basbaum, 2009; Grundy et al., 2019). However, unlike skin, pain sensations arising from the gut (visceral pain) are poorly localized and poorly defined. There is also an inability in most parts of the gut, particularly the intestine and colon, to discriminate between different types of pain, e.g., temperature (cold versus heat pain) from mechanical distension-induced pain. Regardless of the type of gut pain, what remains mysterious is what type(s) of sensory neuron(s) may encode pain sensations. That is, which neurons are nociceptive and which ones encode non-nociceptive stimuli? There is some evidence that the vagus nerve can encode some noxious stimuli from the upper gut (Ru et al., 2011). However, of the two major sensory nerves that innervate the gut wall (vagal afferents or spinal afferents), there is an overwhelming body of evidence to suggest that sensory neurons arising from spinal dorsal root ganglia (DRG) are the major sensory nerves encoding noxious stimuli from the gut to the brain (Basbaum, 2009; Brierley et al., 2018). Recent studies have shown that by surgically removing specific populations of DRG, pain reflex responses to mechanical distension of the colon can be ablated (Kyloh et al., 2022). This occurs whilst leaving all other sensory and motor nerves intact (Kyloh et al., 2022).

## Which spinal afferents contribute to the sensation of pain?

Extracellular recordings have been made from spinal afferents that lie between the colon and spinal cord (Brierley et al., 2004, 2005a; Feng et al., 2010, 2012; Feng and Gebhart, 2011; Zagorodnyuk et al., 2011, 2012) and intracellular recordings from spinal afferent neurons in DRG that directly communicate with intact colon in *ex vivo* preparations (Malin et al., 2009; Hibberd et al., 2016). When extracellular recordings are made from colonic afferents five distinct classes of afferent fiber can be distinguished, based on mechanical stimuli using von Frey hair probing and/or stretch (Brierley et al., 2004). Intracellular recordings from the nerve cell bodies of colon-projecting spinal afferents has revealed primarily high and low threshold firing afferents (Malin et al., 2009; Hibberd et al., 2016) many of which are peptidergic and express the alpha isoform of the calcitonin gene related peptide (CGRPα) (Hibberd et al., 2016). Interestingly, when electrophysiological recordings have been made from colon-projecting spinal afferents, the vast majority have been shown to generate action potentials following distension at low thresholds, referred to as low responders (Malin et al., 2009; Hibberd et al., 2016). And, these axons are exclusively C-fibers (Malin et al., 2009), with low conduction velocities. Another study found similar findings (Hibberd et al., 2016), in that most colon-projecting DRG neurons are activated at low mechanical distension thresholds and over a wide-dynamic range, that includes the both innocuous (non-painful) stimuli and noxious levels of distension pressure (Hibberd et al., 2016).

Retrograde tracing from DRG revealed that >80% of thoracolumbar DRG neurons express TRPV1 (Robinson et al., 2004; Brierley et al., 2005b) and 50% of retrogradely labeled lumbosacral DRG neurons that project to colon express TRPV1 (Brierley et al., 2005b). TRPV1 expression occurred in primarily low frequency responding neurons (87% were TRPV1 +) while only 13% of high frequency responding neurons were TRPV1 + (Malin et al., 2009). When the nerve endings of spinal afferents have been identified in the colon, that vast majority are peptidergic (CGRPα +) and express TRPV1 (Sharrad et al., 2015).

In the gut, only recently have we been able to visualize with clarity the morphological features of the different types of the nerve endings of spinal afferents by anterograde tracing from DRG (Spencer et al., 2016, 2020a,b). Using this method, spinal afferent endings have been identified in myenteric ganglia as intraganglionic varicose endings (Spencer et al., 2014). On rare occasions intraganglionic lamellar endings (IGLEs) can arise from spinal afferents with cell bodies in DRG (Spencer et al., 2014). In the skin, “free nerve endings” are unspecialized bare nerve endings that lack encapsulation at their terminals and lack complex morphologies (Handler and Ginty, 2021). Whilst spinal afferent nerve endings in the gut are also unencapsulated (similar to free-endings in skin), they rarely exhibit simple morphologies (Spencer et al., 2014). In fact, spinal afferent endings in the gut exhibit an extraordinarily complex array of different morphologies. Recently, we identified that a single DRG neuron can give rise to different morphological types of nerve ending in different anatomical layers of the gut wall (Spencer et al., 2020a,b). Hence, assigning the nomenclature of “free-ending” for all visceral afferents is a broad misnomer.

## Can we distinguish intrinsic sensory nerve endings from extrinsic sensory nerve endings?

A major complication with understanding the extrinsic sensory innervation of the gut, is that the gut itself is unique and has evolved not only with its own nervous system, (known as the enteric nervous system, ENS) (Furness, 2006; Spencer and Hu, 2020), but also its own intrinsic sensory neurons (Kunze et al., 1995; Spencer and Smith, 2004), that express the beta isoform of the calcitonin gene relate peptide (CGRPβ) (Mulderry et al., 1988; Furness et al., 2004; Hibberd et al., 2022). However, it is currently not possible to use CGRP antisera to differentiate the nerve endings of intrinsic sensory neurons from extrinsic sensory nerves. It is also unclear whether intrinsic sensory neurons provide any collateral projections onto the nerve endings of spinal afferents that also innervate myenteric ganglia. Interestingly, it has been proposed that intrinsic sensory neurons in the myenteric plexus may interact with vagal afferent terminals in the gut-brain axis (Perez-Burgos et al., 2014).

## Can we identify nociceptors in the gut using antibodies?

The problem with using terminology like “visceral nociceptors” in the gut is that well accepted nociceptive ion channels, like TRPV1 are highly expressed in the vast majority (∼90%) of spinal afferent neuron that innervate the colon (Malin et al., 2009). In this regard, in both the colon and bladder, the definition of a visceral afferent as a “nociceptive neuron” based solely on expression of TRPV1 is unhelpful. From our experience, there is no neurochemical marker to date, that can discriminate between different populations of spinal afferent nerve endings in the gut, let alone nerve endings that maybe nociceptors. While it is well accepted that nociception from the gut involves TRPV1 + primary afferent neurons (Jones et al., 2005), it is unclear whether a single neurochemical class of TRPV1 + nociceptor or single morphological class of spinal afferent ending exclusively encodes nociception from the gut. This seems highly unlikely, given the fact that most spinal afferents to the colon are peptidergic (Spencer et al., 2014) and express Trvp1 (Sharrad et al., 2015) and that low levels of stretch to the colon activate spinal afferents that indeed also respond to capsaicin (Spencer et al., 2008).

## Can we identify nociceptors in the gut by using morphological studies of transgenic reporter mice?

Transgenic reporter mice have been useful for bulk labeling of large populations of axons in peripheral organs, like the gut. However, they have not been useful for identifying the detailed morphology of the nerve endings of putative “nociceptors” in any visceral organs. This is because firstly, many of the neurochemical markers expressed in spinal afferents are also expressed in vagal afferents or enteric neurons, making interpretation of the origin of the cell bodies of the labeled axons uncertain. And, as mentioned, in reporter mice, labeling of so many axons precludes identification of the fine morphological features that may arise from a single primary afferent. The use of intersectional neurogenetics to narrow the targeted populations in transgenic mice by excluding enteric neuronal populations may overcome this issue in the coming years as the approach becomes more popular. However, all reporter mice will likely label numerous axons that overlap each other making it very difficult to resolve endings that arise from one primary afferent neuron.

## Identifying spinal afferent nerve endings using anterograde tracing from DRG

Anterograde technique from single DRG in live mice has enabled unequivocal identification of spinal afferent nerve endings (Spencer et al., 2014). Using this approach, the spinal afferent endings have now been identified in bladder (Spencer et al., 2018), colon (Spencer et al., 2014), stomach (Spencer et al., 2016), bone (Thai et al., 2020), and uterus (Dodds et al., 2021). What became immediately clear from these studies in gut was that there was a far greater complexity and diversity of different types of endings than we expected. It was also clear that a single DRG neuron did not give rise to sensory endings in only one layer of the gut. In fact, tracing from single DRG neurons revealed that single afferents that projected to the colon had nerve terminal endings in the circular muscle and myenteric ganglia (Spencer et al., 2020b) and another population gave off nerve endings in the myenteric ganglia, submucosa and mucosa (Spencer et al., 2020a). Other classes have yet to be identified using this technique.

## Surgically removing DRG from animals to identify pain pathways

In a recent study, a survival surgical technique was developed to remove specific populations of DRG unilaterally or bilaterally, from live mice (Kyloh et al., 2022). This approach had the major advantage of not interfering with any other afferent of efferent nerve pathways, only spinal afferents, in live mice. When the lumbosacral DRGs (L5-S2) were surgically removed from mice using this approach, the distension-evoked pain pathway (VMR) from the rectum was abolished (Kyloh et al., 2022). This revealed important information that although thoracolumbar DRG innervate (to a small degree) the rectum, the functional pain reflex response (VMR) to rectal distension is abolished if the lumbosacral DRG are removed, but thoracolumbar DRG are retained (Kyloh et al., 2022). This is significant because the lumbosacral DRG pathway to the rectum in mice is primarily a low threshold, wide dynamic range sensory pathway.

Taken together, it is suggested that there may well be no exclusive nociception-dedicated primary afferent neuronor transduction site(s) in the viscera. Rather, nociception may arise from bulk signaling across all visceral afferent morphological and neurochemical subclasses. Indeed, stimuli such as noxious distension that may recruit afferents with high-distension thresholds inevitably recruit all low-threshold afferents too, which can continue to encode higher firing intensities into the noxious range.

## Conclusion

As more scientists are turning their attention to understanding gut-brain communication *via* primary sensory nerves, it is important we develop a clearer understanding of which class(es) of afferent fibers that lie between the gut and brain may contribute to nociception. This is an essential step for development of novel therapeutics to potentially target visceral (gut) nociception. The discovery large populations of TRPV1 expressing spinal afferent neurons underlie a major component of the mechanically activated pain pathway from colon to brain suggests that low threshold mechanically activated ion channels play major roles in encoding both physiological (non-noxious) and noxious pain signals to the brain. What is clear is that there is an extraordinarily complex array of different morphological types of spinal afferent ending in the gut; and it is becoming increasingly unlikely that a single population of sensory neuron underlies nociception and pain-signaling along the gut-brain axis.

## Data availability statement

The original contributions presented in this study are included in the article/supplementary material, further inquiries can be directed to the corresponding author.

## Author contributions

NS wrote the manuscript in collaboration with TH, ZX, and HH. All authors corrected and approved the final manuscript.
